# Hyperproduction of poly(4-hydroxybutyrate) from glucose by recombinant *Escherichia coli*

**DOI:** 10.1186/1475-2859-11-54

**Published:** 2012-05-02

**Authors:** Xiao-Yun Zhou, Xiao-Xi Yuan, Zhen-Yu Shi, De-Chuang Meng, Wen-Jun Jiang, Lin-Ping Wu, Jin-Chun Chen, Guo-Qiang Chen

**Affiliations:** 1 Department of Biological Science and Biotechnology, MOE Key Lab of Bioinformatics and Systems Biology, School of Life Sciences, Tsinghua-Peking Center for Life Sciences, Tsinghua University, Beijing 100084, China; 2 Department of Chemistry, University of Melbourne, Parkville, VIC 3052, Australia; 3 Department of Pharmaceutics and Analytical Chemistry, Faculty of Pharmaceutical Sciences, University of Copenhagen, Copenhagen, Denmark; 4 Center for Nano and Micro Mechanics, Tsinghua University, Beijing 100084, China

**Keywords:** Poly(4HB), PHB, Polyhydroxyalkanoates, PhaP, 4-hydroxybutyrate, *Escherichia coli*, Metabolic engineering, Synthetic biology

## Abstract

**Background:**

Poly(4-hydroxybutyrate) [poly(4HB)] is a strong thermoplastic biomaterial with remarkable mechanical properties, biocompatibility and biodegradability. However, it is generally synthesized when 4-hydroxybutyrate (4HB) structurally related substrates such as γ-butyrolactone, 4-hydroxybutyrate or 1,4-butanediol (1,4-BD) are provided as precursor which are much more expensive than glucose. At present, high production cost is a big obstacle for large scale production of poly(4HB).

**Results:**

Recombinant *Escherichia coli* strain was constructed to achieve hyperproduction of poly(4-hydroxybutyrate) [poly(4HB)] using glucose as a sole carbon source. An engineering pathway was established in *E. coli* containing genes encoding succinate degradation of *Clostridium kluyveri* and PHB synthase of *Ralstonia eutropha.* Native succinate semialdehyde dehydrogenase genes *sad* and *gabD* in *E. coli* were both inactivated to enhance the carbon flux to poly(4HB) biosynthesis. Four PHA binding proteins (PhaP or phasins) including PhaP1, PhaP2, PhaP3 and PhaP4 from *R. eutropha* were heterologously expressed in the recombinant *E. coli,* respectively, leading to different levels of improvement in poly(4HB) production. Among them PhaP1 exhibited the highest capability for enhanced polymer synthesis. The recombinant *E. coli* produced 5.5 g L^-1^ cell dry weight containing 35.4% poly(4HB) using glucose as a sole carbon source in a 48 h shake flask growth. In a 6-L fermentor study, 11.5 g L^-1^ cell dry weight containing 68.2% poly(4HB) was obtained after 52 h of cultivation. This was the highest poly(4HB) yield using glucose as a sole carbon source reported so far. Poly(4HB) was structurally confirmed by gas chromatographic (GC) as well as ^1^H and ^13^C NMR studies.

**Conclusions:**

Significant level of poly(4HB) biosynthesis from glucose can be achieved in *sad* and *gabD* genes deficient strain of *E. coli* JM109 harboring an engineering pathway encoding succinate degradation genes and PHB synthase gene, together with expression of four PHA binding proteins PhaP or phasins, respectively. Over 68% poly(4HB) was produced in a fed-batch fermentation process, demonstrating the feasibility for enhanced poly(4HB) production using the recombinant strain for future cost effective commercial development.

## Background

A large variety of bacteria are able to accumulate diverse polyhydroxyalkanoates (PHA) as intracellular carbon and energy storage material under nutritional unbalanced conditions [1-4]. Due to their diverse structures, chirality, biodegradability and biocompatibility, PHA have attracted attentions from academic and industrial communities for their potential applications in areas of agriculture, medicine, and materials [2,5-7]. More than 150 types of hydroxyalkanoic acids have been known as monomers of PHA, leading to diverse polymer physical properties [8-11]. Some of the PHA monomers and oligomers were reported to stimulate cell proliferations [12,13].

Homopolyesters of 4-hydroxybutyrate, or Poly(4HB), is a strong thermoplastic material with an elongation to break of 1000%, which means it can be stretched 10 times its original length before it is broken [14]. Due to the remarkable mechanical properties, biocompatibility and biodegradability, poly(4HB) has been approved by the United States Food and Drug Administration (FDA) as the first PHA medical implant material among several PHA materials under investigation [5,15,16].

4-hydroxybutyrate (4HB) was first detected in a copolyester of 3HB and 4HB isolated from *R. eutropha *[17] and generally the incorporation of 4HB into PHA occurred only if 4-Hydroxybutyrate, 4-butyrolactone, 1,4-butanediol or 4-chlorobutyrate was provided as carbon source [18]. Poly(4HB) homopolyester was first reported to be synthesized by *R. eutropha* using 4-hydroxybutyrate as a precursor [19]. Wild-type bacteria like *Comanonas acidovorans *[20] and *Hydrogenophaga pseudovorans *[21] were also found to produce poly(4HB). Recombinant *E. coli* expressing *R. eutropha* PHA synthase gene *phaC* and *Clostridium kluyveri* 4HB-CoA:CoA transferase gene *orfZ* were found able to synthesize poly(4HB) homopolyester when both glucose and 4HB were provided as carbon sources [22,23]. As a general rule, structurally related substrates of 4HB such as γ-butyrolactone, 4-hydroxybutyrate or 1,4-butanediol (1,4-BD) are required as precursors for poly(4HB) synthesis [24]. However, these substrates are much more expensive than glucose, leading to the high cost of poly(4HB) production. Song et al. succeeded in producing poly(4HB) homopolyester using glucose as a sole carbon source in recombinant *E. coli *[25]. However, the reported yield (0.78 g L^-1^) was low for mass cultivation. The high cost of raw material and the low yield of poly(4HB) prevents wide exploitation of poly(4HB) for more applications [26].

**Figure 1:**
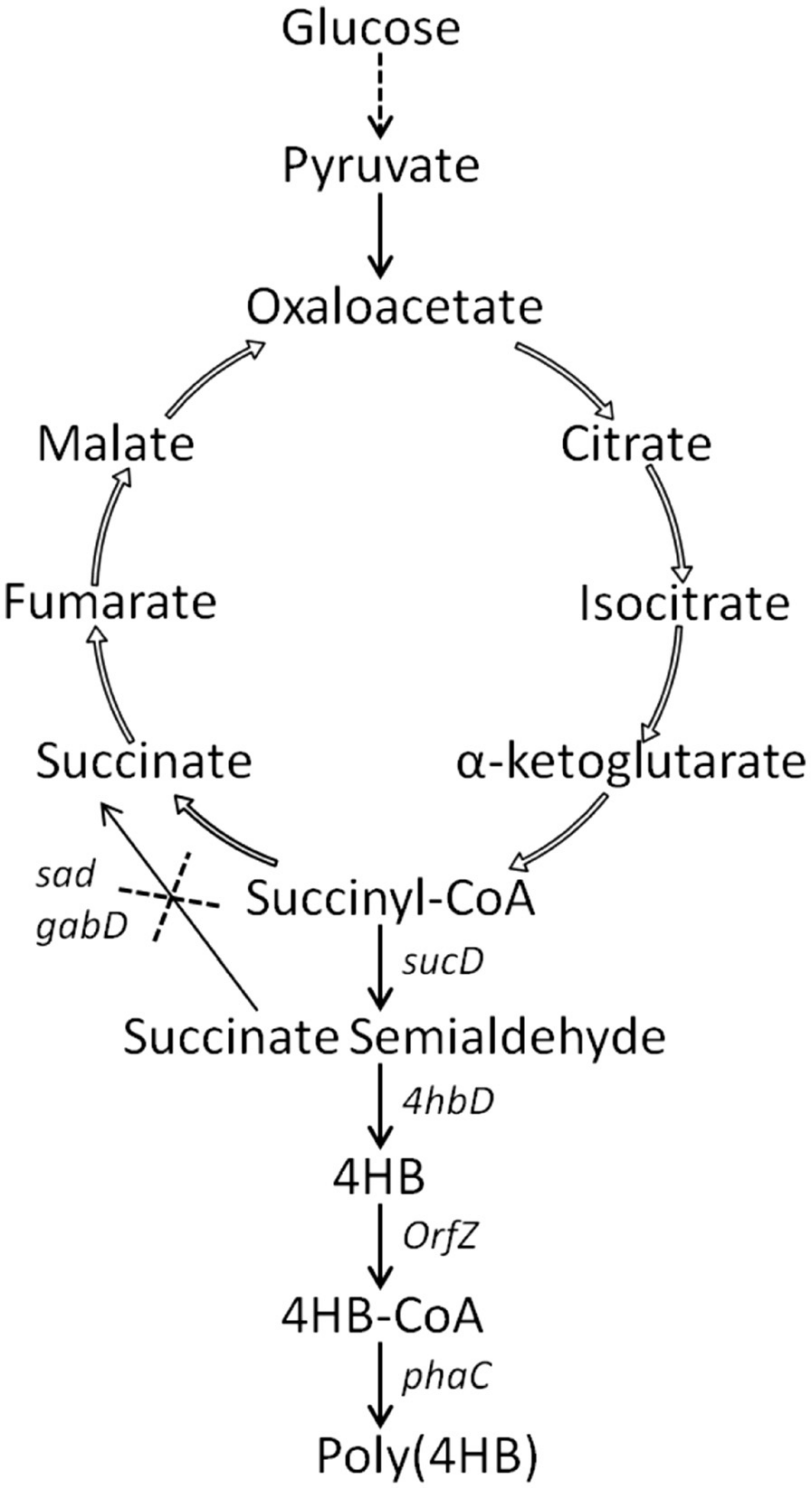
**Engineering pathway for microbial production of Poly(4HB) from glucose in*E. coli*. **Enzymes encoded by the over-expressed genes in the pathway: *sucD*, succinate semialdehyde dehydrogenase of *C. kluyveri*; *4hbD *, 4-hydroxybutyrate dehydrogenase; *orfZ*, 4HB-CoA:CoA transferase; *phaC*, PHA synthase; *sad* and *gabD*, succinate semialdehyde dehydrogenase of *E. coli*.

Studies on producing poly(3-hydroxybutyrate-*co*-4-hydroxybutyrate) or P3HB4HB from glucose in *E. coli* were reported [27,28]. The 4HB monomer was synthesized from anaerobic succinate degradation pathway of *C. kluyveri*. In this pathway, the intermediate of tricarboxylic acid (TCA) cycle succinyl-CoA was converted to succinate semialdehyde (SSA) by SSA dehydrogenase, and SSA was reduced to 4HB by 4HB dehydrogenase (Figure 1) [29,30]. Subsequently, 4HB was converted to 4HB-CoA via 4HB-CoA:CoA transferase. In *C*. *kluyveri*, these three enzymes were encoded by genes *sucD4hbD* and *orfZ*, respectively [28].

*E. coli* possesses two forms of SSA dehydrogenase (SSADH) encoded by *sad* and *gabD* first identified in *R. eutropha *[31], both SSADH catalyze degradation of SSA to succinate which can reduce the SSA flux to 4HB-CoA pathway. Li et al. reported an enhancement on 4HB content in P3HB4HB when SSADH was inactivated in *E. coli*, indicating a possibility of producing poly(4HB) in *sad* and *gabD* deficient mutant.

Phasins are small amphiphilic proteins localizing at the surface of PHA granules and there are interactions among various phasins [32-35]. They play important roles in PHA synthesis and granule formation [36]. The PhaP phasins were proven to promote PHB synthesis by regulating the surface/volume ratio of PHB granules or by interacting with PHA synthase yet without influencing PHA molecular weights [37]. Four genes encoding highly homologous phasins including phaP1, phaP2, phaP3 and phaP4 were found in *R. eutropha*, among which PhaP1 is the major phasin protein [38-40].

The aim of this study was to construct a recombinant *E. coli* for hyperproduction of poly(4HB) using glucose as a sole carbon source.

## Results

### Synthesis of poly(4HB) by recombinant *E. coli* grown in shake flasks

**Figure 2:**
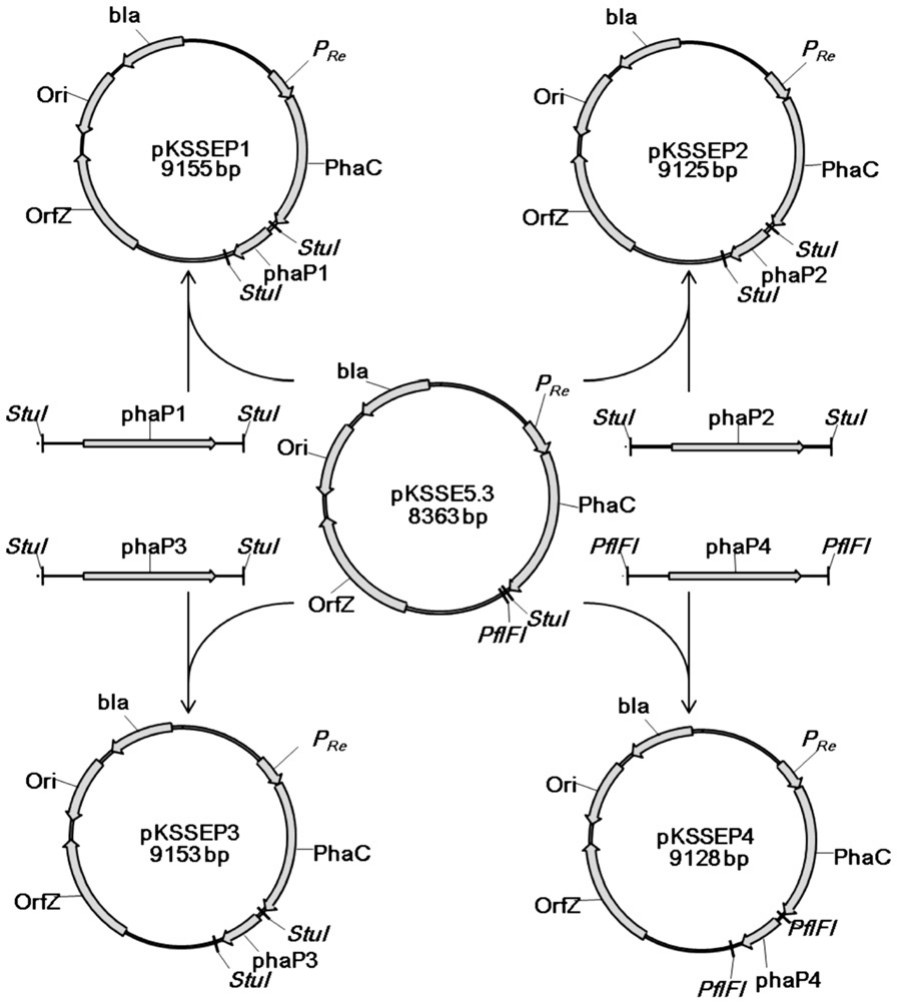
Constructions of plasmids used in this study.

Biosynthesis pathway of poly(4HB) was constructed in *sad* and *gabD* deficient strain *E. coli* JM109SG by co-expressing *sucD*, *4hbD*, *orfZ* and *phaC* heterologously using compatible plasmids pMCSH5 harboring *sucD* and *4hbD* and pKSSE5.3 harboring *orfZ* and *phaC* (Figures 1 and 2). To study the function of PhaP on poly(4HB) production, four plasmids pKSSEP1, pKSSEP2, pKSSEP3 or pKSSEP4 were co-transformed with the plasmid pMCSH5 into *E. coli* JM109SG, respectively. In the pKSSEPx plasmid series, genes *phaC* and *phaPx* shared the same promoter P_Re_ from *R. eutropha* while *orfZ* gene was initiated by its own promoter (Figure 2). In plasmid pMCSH5, *sucD* and *4hbD* genes were controlled by promoter P_*pdc*._ The function of poly(4HB) biosynthesis pathway with or without PhaP was tested in *E. coli* JM109 and its SSADH deficient strain was cultivated in shake flasks for 48 h in LB medium supplemented with 20 g L^-1^ glucose and PBS buffer.

Gas chromatographic analysis of derivatives obtained from lyophilized cells provided the single peak representing the methyl ester of 4HB, demonstrating that the resulting PHA was a poly(4HB) homopolyester. As expected, *E. coli* JM109 (pKSSE5.3, pMCSH5) did not produce any polyester. In comparison, its *sad* and *gabD* deficient mutant *E. coli* JM109SG (pKSSE5.3, pMCSH5) grew to 3.8 g L^-1^ CDW containing 12 wt% poly(4HB) (Table 1). The co-expression of PhaP_1-4_ in *E. coli* JM109SG (pKSSE5.3, pMCSH5) led to enhancements of poly(4HB) accumulation from 12 wt% without any PhaP to at least 22 wt% with PhaP4 to a maximum of 35 wt% with PhaP1. CDW also reached the highest of 5.5 g L^-1^ containing more than 35 wt% poly(4HB) when *phaP1* was expressed in *E. coli* JM109SG (pKSSEP1, pMCSH5). Expression of *phaP3* resulted in second highest poly(4HB) accumulation of 32 wt% CDW by *E. coli* JM109SG (pKSSEP3, pMCSH5). While PhaP2 and PhaP4 showed a similar lower ability on the improvement of poly(4HB) synthesis by the *E. coli*. The results were consistent with the different roles played by the four PhaP phasins for PHA synthesis [39,40].

**Table 1:** **Shake flasks study of poly(4HB) production from glucose by *E. coli *strains grown in shake flasks**^**a**^

***E. coli***	**Plasmids**	**CDW^*b*^(g L^-1^)**	**Poly(4HB) content^*c*^ (wt%)**	**Poly(4HB) (g L^-1^)**
JM109	pKSSE5.3, pMCSH5	3.33±0.13	-^d^	-
JM109SG	pKSSE5.3, pMCSH5	3.83±0.15	12.13±0.53	0.47±0.04
JM109SG	pKSSEP1, pMCSH5	5.46±0.04	35.39±0.80	1.93±0.06
JM109SG	pKSSEP2, pMCSH5	4.30±0.17	22.85±1.06	0.98±0.07
JM109SG	pKSSEP3, pMCSH5	4.28±0.12	32.35±1.42	1.39±0.09
JM109SG	pKSSEP4, pMCSH5	4.34±0.15	22.95±2.58	1.00±0.14

^*a*^ Cells were cultivated in LB medium at 37°C and 200 rpm for 48 h as described in “Materials and methods”

20 g L^-1^ glucose and PBS buffer were added to the medium after sterilization. Three parallel studies were conducted for each data.

^***b***^ CDW: cell dry weight.

^***c***^ Poly(4HB) content: PHA contents are given as mass percentage of CDW.

^d^ Not detected.

### Production and structure confirmation of poly(4HB) from fermentor study

**Figure 3:**
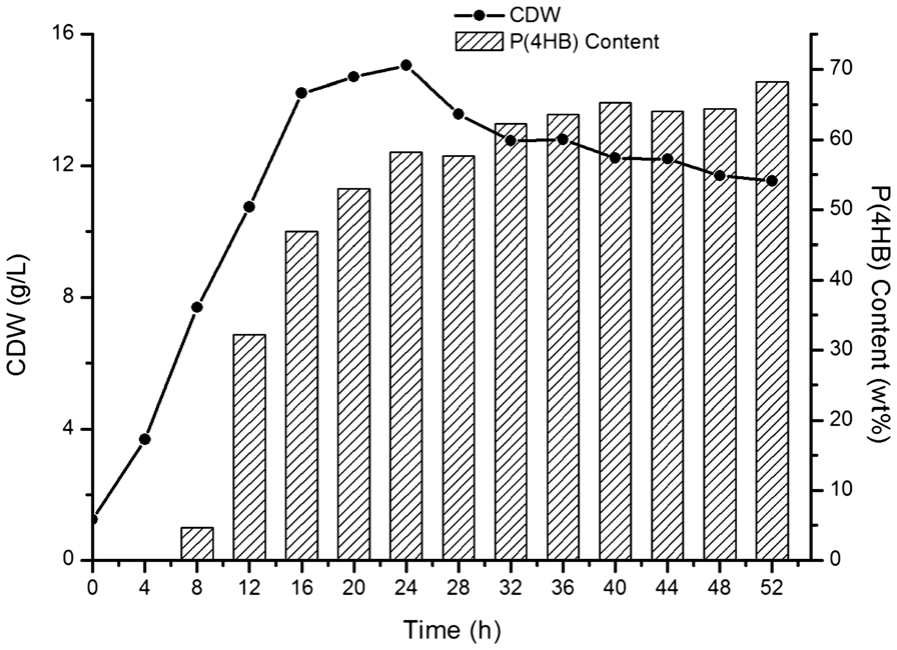
**Growth and poly(4HB) production by recombinant*E. coli* harboring pKSSEP1 and pMCSH5 cultivated in a 6-l fermentor.**

As revealed by the shake flask results (Table 1), *E. coli* JM109SG (pKSSEP1, pMCSH5) showed the fastest growth rate and highest poly(4HB) accumulation level among all strains studied. It was therefore selected for further studies using well-controlled fermentor. *E. coli* JM109SG (pKSSEP1, pMCSH5) was grown in the modified LB medium containing tripled amount of yeast extract in a fed-batch fermentation process maintaining 10 g L^-1^ glucose during the entire period. After 52 h of fermentor cultivation, the cells grew to approximately 12 g L^-1^ CDW containing over 68 wt% poly(4HB) in the expense of a total of 90 g L^-1^ glucose consumed. This was by far, the highest poly(4HB) production using glucose as a sole carbon source. The poly(4HB) synthesis increased very fast during the exponential growth phase, and reached a relatively stable level at over 60 wt% poly(4HB) in CDW after 32 h (Figure 3).

**Figure 4:**
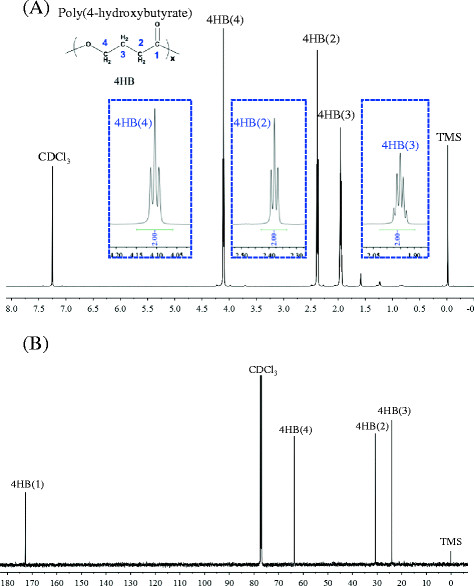
^**1**^**H NMR (A) and**^**13**^**C NMR spectra (B) of poly(4HB).** Numbering scheme were the same as that of poly(4HB) molecular structure described in (A). The inserted dashed enveloped areas in (A) were the enlarged details of each proton split peaks. Chemical shifts were in ppm and tetramethylsilane (TMS) was employed as an internal chemical shift standard.

To confirm homopolyester structure, gas chromatography and NMR were employed. As evidenced by spectra of ^1^ H NMR and ^13^ C NMR (Figure 4), three well-characterized proton resonances, namely, 4HB (2): δ 2.39-2.37 ppm, 4HB (3): δ 1.97-1.93 ppm, 4HB (4): δ 4.12-4.09 ppm, appeared with identical intensities assigned to only 4HB units (Figure 4A). In the inserted dashed chart, the protons of 4HB (2) and 4HB (4) were found split into three peaks, while the peak of 4HB (3) proton was quadrupled based on the “N +1 rule”: a proton with N neighbors appears as a cluster of N + 1 peaks. For example, the proton of 4HB (3) is neighbored to 4HB (2) and 4HB (4), and it has four protons around it as shown from the molecular structure of 4HB, thus, the proton of 4HB (3) is split into five peaks (Figure 4A). Furthermore, the four ^13^ C resonances at 20–180 ppm could be assigned to specific carbon species of 4HB units (Figure 4B). The carbon of carbonyl group 4HB (1) had the highest field in ^13^C NMR, the chemical shift was δ 172.74 ppm. From left to right, the chemical shifts of δ 63.61 ppm, δ 30.73 and δ 24.74 ppm belonged to 4HB (4), 4HB (2) and 4HB (3), respectively. Based on these data, the NMR spectra confirmed the polyester sample be a homopolyester consisting of only 4-hydroxybutyrate.

### Physical characterization of poly(4HB) produced by the recombinant *E. coli*

Poly(4HB) produced by *E. coli* JM109SG (pKSSEP1, pMCSH5) was extracted and purified before casting into films for mechanical property studies. The white and foggy poly(4HB) films exhibited a much higher elasticity than other known PHA. Its elongation at break (ε_b_), tensile strength (σ_t_), and Young’s modulus (E) was 1014%, 32.55 MPa and 12.8 MPa, respectively. The poly(4HB) had a molecular mass of weight average molecular weigh (M_w_) of 0.22 × 10^6^ together with a polydispersity (M_w_/M_n_) of 2.11 as determined by gel-permeation chromatography.

## Discussion

As a strong pliable thermoplastic material with good flexibility, poly(4HB) has been approved by FDA as a suture material (http://www.tepha.com). Biomedical applications are usually not sensitive to high cost. However, a reduction on poly(4HB) production cost should allow for more application exploitation. High production cost for poly(4HB) comes from expensive 4HB precursors including 4-hydroxybutyric acid, γ-butyrolacton or 1,4-butanediol [41,42], and from very low yield of poly(4HB) by recombinant bacteria. Therefore, simple and low cost substrates as well as a highly productive strain can help reduce poly(4HB) production cost.

The anaerobic succinate degradation pathway employed in this study conferred on the recombinant *E. coli* the ability to utilize glucose as a sole carbon source for poly(4HB) production. The additional expression of PHA granule associate protein PhaP provided a further enhancement on poly(4HB) yield, allowing for further fermentor exploitation. While in wild *E. coli* strain, succinate semialdehyde can be degraded to succinate by SSA dehydrogenase (SSADH) encoded by *sad* and *gabD*[30]*,* leading to a decreased metabolic flux to 4HB production (Figure 1). To channel more flux to 4HB, the native SSADH genes of *E. coli* were inactivated in the poly(4HB) producing recombinant. Shake flask studies of *E. coli* JM109 and its SSADH deficient mutant JM109SG harboring pMCSH5 and pKSSE5.3 showed that inactivation of SSADH genes significantly improved poly(4HB) synthesis compared with the wild strain JM109 which had no poly(4HB) production at all (Table 1). On the other hand, the highest 4HB molar fraction in P3HB4HB synthesized from glucose in *E. coli* was 11% reported so far [28]. Our result indicated that the recombinant enzymes in this pathway were active enough to provide sufficient 4HB from glucose for polymerization.

Expression of all four PhaPs (phasin) cloned from *R. eutropha* provided additional improvement on poly(4HB) accumulation in the order of PhaP1 > PhaP3 > PhaP2 > PhaP4 (Table 1). The differences of their different influences are not clear yet but probably due to the different roles of PhaP played on PHA granules formation. PhaP1 was the major phasin with the highest expression amount in *R. eutropha* while PhaP2, PhaP3 and PhaP4 were small in quantity [39,40], indicating its dominating function for PHA granule formation, and PhaP3 was expressed at a significantly high level in PhaP1 deficient strains, other PhaPs were in much lower levels. Our results therefore suggested that the poly(4HB) yields were positively related to the expression levels of PhaP.

The recombinant *E. coli* JM109SG (pKSSEP1, pMCSH5) grown to 12 g L^-1^ CDW under a well-controlled fermentor run in a fed-batch process accumulated over 68% poly(4HB) using glucose as the only carbon source over a 52 h period (Figure 3). This is by far the highest yield for poly(4HB). In its exponential growth period of 8–24 h after innoculation, poly(4HB) content increased most rapidly and reached a relatively stable level when cells entered the stationary phase. As in the exponential phase, TCA cycle is most active, supplying the most succinyl-CoA for the poly(4HB) synthesis, leading to a rapid poly(4HB) accumulation rate. A continuous fermentation process that maintains the cells in their exponential growth phase may further improve poly(4HB) accumulation level.

## Conclusion

In summary, *Escherichia coli* strain JM109 harboring an engineering pathway encoding succinate degradation genes of *Clostridium kluyveri* and PHB synthase gene of *Ralstonia eutropha* together with its native succinate semialdehyde dehydrogenase genes *sad* and *gabD* inactivated, was able to achieve significant level of poly(4HB) biosynthesis from glucose. Additional expression of four PHA binding proteins PhaP or phasins in the recombinant strain, respectively, led to a further improvement of poly(4HB) accumulation. PhaP1 was found most useful among the four PhaPs used. Over 68 wt% poly(4HB) was produced in a fed-batch fermentation process, demonstrating the feasibility for enhanced poly(4HB) production using the recombinant strain for future cost effective commercial development.

## Methods

### Bacterial strains and plasmids

The bacterial strains and plasmids used in this study were listed in Table 2. *E. coli * Trans1-T1 from TransGen Biotech (Beijing, China) was used for plasmids construction. *Ralstonia eutropha* was used as a template for gene cloning [43]. *E. coli* JM109 (TaKaRa, Dalian, China) and its *sad* and *gabD* deficient strain *E. coli* JM109SG were used for gene expression and poly(4HB) accumulation.

**Table 2:** Bacterial strains and plasmids used in this study

**Name**	**Relevant characteristics**	**Source or reference**
**Strains**		
*E. coli* JM109	*recA1, endA1, gyrA96, thi, hsdR17, supE44, relA1, Δ(lac proAB)/F’*	TaKaRa (Dalian, China)
	[*traD36, proAB*^*+*^*, lac*^*q*^*lacZΔ*M15]	
*E. coli* JM109SG	JM109 ∆*sad ∆gabD*	[28]
*E. coli* Trans1-T1	The fastest growing chemically competent strain currently available	TransGen Biotech (Beijing, China)
*Ralstonia eutropha* H16	Wild type	ATCC17699^a^[43]
**Plasmids**		
pKSSE5.3	pBluescript vector derived, containing *phaC* and *orfZ,* Amp^R^	[22]
pKSSEP1	phaP1 gene inserted into pKSSE5.3, Amp^R^	This study
pKSSEP2	phaP2 gene inserted into pKSSE5.3, Amp^R^	This study
pKSSEP3	phaP3 gene inserted into pKSSE5.3, Amp^R^	This study
pKSSEP4	phaP4 gene inserted into pKSSE5.3, Amp^R^	This study
pMCSH5	*sucD*-*4hbD* inserted into pBBR1MCS-2, Km^R^	[28]
**Primers (5 ′ → 3′)**		
phaP1F	AGTCTAGGCCTAAGAAATGCGCCTTGACCCACCC	This study
phaP1R	AGTCTAGGCCTGCAAAACACACCGCAAACGCCAG	
phaP2F	CAGCGAGGCCTGTTCGCAATGCTGCAATCTTTATT	This study
phaP2R	ACTATAGGCCTATACCACCCGTGACAACGGCAAG	
phaP3F	ACTATAGGCCTGATTCGCACTCGGATGCTGCGCT	This study
phaP3R	CAGCGAGGCCTTTGTATACCGATGCGGGAAGATT	
phaP4F	CAGCGGACGTTGTCTCACGATGCAGCAATTGTTTTCC	This study
phaP4R	AGTCTGACGTTGTCCTTCGACACGAAGGAAGTTTAGGC	

^a^ American Type Culture Collection.

Plasmid pKSSE5.3 was constructed by Hein et al. containing genes *phaC* and *orfZ *[22]. Plasmids pKSSEPx, with “x” referring to 1, 2, 3 and 4 in this study, were constructed by subcloning genes *phaP1, phaP2, phaP3 and phaP4* from genome of *R. eutropha,* followed by inserting them into the plasmid pKSSE5.3, respectively. Primers phaPxF and phaPxR were used for PCR amplification. The PCR products of *phaP1phaP2* and *phaP3* fragments were digested by *StuI* while *phaP4* fragment digested by *pFlfI*. The gel electrophoresis-purified fragments were then ligated with pKSSE5.3 digested by the respective endonuclease (Figure 2). Plasmid pMCSH5 was constructed by Li et al. containing *sucD* and *4hbD* genes [28].

### Cultivation conditions and culture medium

Plasmids pKSSE5.3 or pKSSEPx were co-transformed with pMCSH5 into *E. coli* JM109 and its SSADH deficient mutant by electroporation.

For shake flask cultivations, the growth process was carried out on a rotary shaker at 200 rpm in 500 mL conical flasks containing 50 mL LB medium supplemented with 20 g L^-1^ glucose at an inoculation volume of 4% for 48 h. For fermentation studies, seed culture was inoculated into a 6-L fermentor (NBS3000, New Brunswick, USA) at 10% inoculation volume with an operating volume of 3 L. The fermentation process was carried out at 37°C, pH 7.0 under a dissolved oxygen concentration (DO) of 30% of saturation. For poly(4HB) accumulation, concentrated glucose was complemented when OD_600_ began to increase exponentially to maintain the concentration of glucose above 10 g L^-1^ during the fermentation process.

For shake flask studies, Luria-Bertani (LB) medium supplemented with 20 g L^-1^ glucose in phosphate buffered saline (PBS) solution with a working concentration of 2.31 g L^-1^ KH_2_PO_4_ and 16.42 g L^-1^ K_2_HPO_4_∙3H_2_O to maintain a pH around 7.0. Glucose and PBS were added to the medium after heat sterilization. During the fermentation process, LB medium with 15 g L^-1^ yeast extract without PBS was used to promote cell growth. 50 mg L^-1^ kanamycin and 100 mg L^-1^ ampicillin were added to the medium to maintain stability of the plasmids during the growth processes.

### Analytical methods

Bacterial cultures were harvested by centrifugation at 3000 g for 10 min and then washed with distilled water. The cell dry weight (CDW) was measured after vacuum lyophilization. PHA content and composition were analyzed by gas chromatography (SHIMADZU GC-2014 C, Kyoto, Japan) after methanolysis of lyophilized cells in chloroform with γ-butyrolactone (Sigma-Aldrich) used as standard [44,45].

### PHA extraction and physical characterization

PHA were extracted from the lyophilized cells [42]. In details: 10 mL chloroform was added to 1 g of dry cells in screw-capped tubes. The tubes were maintained at 100°C for 4 h. Subsequently, equal volume of water was added to the tube and the chloroform at the bottom was sucked out and precipitated with an excess of 10 volumes of ice-cold ethanol [46].

The molecular structure of poly(4HB) was studied using nuclear magnetic resonance (NMR). The sample was dissolve in deuterated-chloroform (CDCl_3_) and recorded the ^1^H NMR and ^13^C NMR spectra. The proton (^1^H) NMR was performed on JOEL JNM- ECA 300 NMR spectrophotometer in deuterated chloroform as a solvent, tetramethylsilane (TMS) was used as an internal chemical shift standard. Carbon (^13^C) NMR spectra was measured on 600 MHz spectrophotometer.

To study its mechanical properties, PHA samples were spread into films by the conventional solvent-casting method [47]. The resulting PHA films were cut into rectangle-shaped specimens with a width of 10 mm and a thickness of approximately 120 μm. The stress–strain measurements of films were carried out using an AL-7000 S testing machine (Gotech Testing Machine, China) at room temperature. The speed of the cross-head was 50 mm min^-1^[48]. Molecular weights were obtained via gel permeation chromatography (GPC Spectra System P2000) equipped with a Shimadzu RID-10A detector.

## Competing interests

The authors declare that they have no competing interests.

## Authors’ contributions

XYZ designed the experiments, constructed the plasmids, performed fermentation studies and prepared the manuscript. XXY performed shake flask experiments. ZYS provided suggestions. DCM and WJJ participated in the fermentation process. LPW analyzed the NMR data. JCC and GQC supervised the study. All authors read and approved the final manuscript.
